# Investigation of bone formation using calcium phosphate glass cement in beagle dogs

**DOI:** 10.5051/jpis.2010.40.3.125

**Published:** 2010-06-25

**Authors:** Seung-Bum Lee, Ui-Won Jung, Youna Choi, Otgonbold Jamiyandorj, Chang-Sung Kim, Yong-Keun Lee, Jung-Kiu Chai, Seong-Ho Choi

**Affiliations:** 1 Department of Periodontology, Research Institute for Periodontal Regeneration, Yonsei University College of Dentistry, Seoul, Korea.; 2 Department and Research Institute of Dental Biomaterials and Bioengineering, Yonsei University College of Dentistry, Seoul, Korea.

**Keywords:** Bone substitutes, Calcium phosphates, Osteogenesis

## Abstract

**Purpose:**

Among available biomaterials, bioceramics have drawn special interest due to their bioactivity and the possibility of tailoring their composition. The degradation rate and formulation of bioceramics can be altered to mimic the compositions of the mineral phase of bone. The aim of this study was to investigate the bone formation effect of amorphous calcium phosphate glass cement (CPGC) synthesized by a melting and quenching process.

**Methods:**

In five male beagle dogs, 4 × 4 mm 1-wall intrabony defects were created bilaterally at the mesial or distal aspect of the mandibular second and fourth premolars. Each of the four defects was divided according to graft materials: CPGC with collagen membrane (CM), biphasic calcium phosphate (BCP) with CM, CM alone, or a surgical flap operation only. The dogs were sacrificed 8 weeks post-surgery, and block sections of the defects were collected for histologic and histometric analysis.

**Results:**

There were significant differences in bone formation and cementum regeneration between the experimental and control groups. In particular, the CPGC and BCP groups showed greater bone formation than the CM and control groups.

**Conclusions:**

In conclusion, CPGC was replaced rapidly with an abundant volume of new bone; CPGC also contributed slightly to regeneration of the periodontal apparatus.

## INTRODUCTION

Regenerative treatment of large periodontal bone defects remains a challenge for periodontists. Autologous bone grafting is considered to be the gold standard in reconstructive bone surgery due to its superior osteogenic potential compared to allogenic transplants. However, the availability of autogenous graft material is limited. Graft harvest lengthens operation time and can be associated with adverse effects, such as bleeding, pain, and infection. Allogenic bone material carries the risk of infectious disease transmission, including human immunodeficiency virus or hepatitis [1,2]. Due to these drawbacks, inorganic composites are of special interest as bone substitutes. Synthetic grafts must be composed of a three-dimensional porous material to induce bone formation and osteoconduction.

Calcium phosphates (CP) have been received the most attention as synthetic agents, and they are widely used because of their good biocompatibility and osteointegrative properties [3]. Synthetic hydroxyapatite (HA) is one type of CP and can be anchored to native bone by the establishment of a physicochemical bond with living tissue [4]. Extensive research has been performed on HA over the past 20 years as a leading candidate of CP, and it has been used clinically in various forms due to its osteoconductive properties. Many investigators have reported that HA resulted in improved attachment and pocket reduction in clinical studies [5].

However, the success of these materials is limited primarily due to inappropriate physical properties such as inadequate toughness, low elasticity, low resorbability, and lack of osteogenic properties [6]. Calcium phosphate glass (CPG) materials are known to have osteoconductive characteristics, serving as an active participatory template for the formation of new bone [7]. CPG is bioactive and has a chemical composition very similar to that of the mineral phase of bone. Recently, LeGeros and Lee reported on CPG cement (CPGC) in a CaO-CaF_2_-P_2_O_5_-MgO-ZnO system, where it was observed to promote bone-like tissue formation in vitro [8]. Moreover, CPGC is easily synthesized by a melting and subsequent quenching process and has a low viscosity in the molten state [9]. Accordingly, CPGC can offer stability in the repair of bone defects during the initial 6 weeks. Previous studies have reported that CPGC has more desireable physical properties than BCP [10]. However, the bone formation and periodontal regeneration abilities of CPGC relative to BCP have not been documented.

The objective of this study was to evaluate the bone formation effect of CPGC in a system of CaO-CaF_2_-P_2_O_5_-MgO-ZnO and to determine the influence of CPGC on periodontal regeneration in 1-wall intrabony defects in the mandibles of beagle dogs by comparing CPGC performance with that of BCP.

## MATERIALS AND METHODS

### Animals

Five male beagle dogs (25-30 kg), 18 to 24 months old were used. The animals had intact dentition and a healthy periodontium. Animal selection, management, surgical protocol and preparation followed routines approved by the Institutional Animal Care and Use Committee, Yonsei Medical Center, Seoul, Korea.

### Experimental groups

Four defects were created in each dog, and one defect was assigned to each experimental group as follows: 1) CPGC group: defect filled with CPGC covered by collagen; 2) BCP group: defect filled with BCP covered by collagen; 3) CM group: defect covered by collagen membrane; 4) Control group: non-grafted.

### Graft materials

BCP (Osteon™, Genoss Co. Ltd., Suwon, Korea) is composed of 70% HA and 30% β-tricalcium phosphate (β-TCP). HA coated with β-TCP establishes an interconnected scaffold with a porosity of 300-500 nm.

CPGC was prepared from a CaO-CaF_2_-P_2_O_5_-MgO-ZnO system. Mixed batches were melted in a platinum crucide at 850℃ and poured onto a graphite plate at room temperature as quenched glass was ground in an alumina mortar. Particle size of the powdered sample was 0.08 to 555.7 µm (average size, 167.95 µm). The CPG was mixed with Na_2_CO_3_ powder in NaOH solution to maintain stability of shape and control the setting time. The final CPGC product was amorphous calcium polyphosphate.

CM (CollaTape®, Integra Lifesciences Co., Plainsboro, USA) was fabricated using collagen obtained from bovine deep flexor (Achilles) tendon.

### Surgical procedure

**Figure 1:**
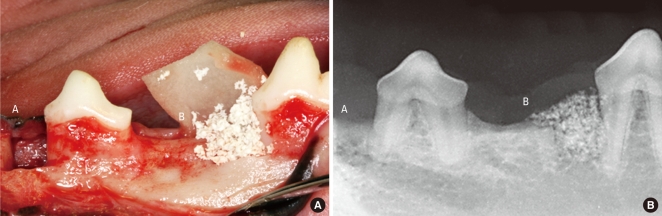
Clinical photograph (A) and radiograph (B) showing surgically prepared 1-wall intrabony defects at the mesial aspect of the second premolar and fourth premolar (A: collagen membrane group or control group, B: calcium phosphate glass cement group or biphasic calcium phosphate).

Surgery was performed under general anesthesia induced by intravenous injection of atropine (0.04 mg/kg) and intramuscular injection of xylazin (Rompun, Bayer Korea, Seoul, Korea) and ketamine (Ketalar, Yuhan Co., Seoul, Korea) followed by the administration of inhaled enflurance. Routine dental infiltration anesthesia (2% lidocaine with HCl epinephrine, 1:100,000, Kwangmyung Pharm., Seoul, Korea) was used at the surgical site. The mandibular 1st and 3rd premolar teeth were extracted in advance of the experimental surgeries, and the extraction sites were allowed to heal for 8 weeks. One-wall intrabony defects measuring 4 × 4 mm were created bilaterally at the mesial aspect of the mandibular 2nd and 4th premolar areas. A reference notch was made with a round bur on the root surface at the base of each defect. In each dog, two defects were filled with CPGC or BCP then covered with CM. One defect was not filled with graft material and was covered with CM. The last defect was treated by surgical flap operation only. The surgical flap was sutured with 5-0 resorbable suture material (Polyglactin 910, braided absorbable suture, Ethicon, Johnson & Johnson Int., Edinburgh, UK). Intramuscular antibiotics were administered for 3 days and a daily dressing of 0.2% chlorhexidine solution (Hexamedin, 2% chlorhexidine, Bukwang Pharm. Co., Seoul, Korea) was applied for 7 days as postsurgical care. The suture was removed after 10 days. The animals were sacrificed 8 weeks after surgery and block sections of the defect sites were collected and prepared for histological and histometric evaluation (Fig. 1).

### Analysis

#### Histologic analysis

The block sections were fixed in 10% buffered formalin and decalcified with 5% nitric acid for 14 days. Paraffin wax blocks were made and sectioned in the mesio-distal direction with a thickness of 4 µm. Each section was stained in hematoxylin and eosin. General histological findings were observed with a stereoscope (Leica DM-LB, Leica, Wetzlar, Germany) and microscope.

#### Histometric analysis

**Figure 2:**
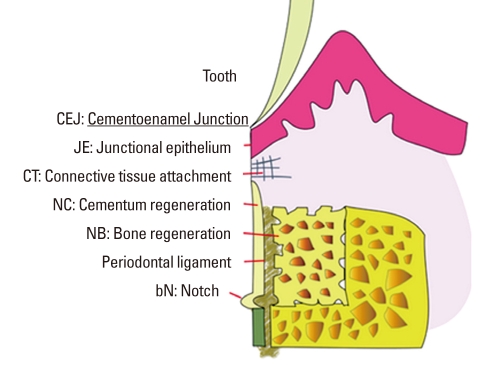
Schematic diagram depicting the landmarks and the parameters used in histometric analysis. The heights of new bone, new cementum, epithelial, and connective tissue attachment in 4 × 4 mm 1-wall intrabony defects were measured using an automated image analysis system.

After conventional microscopic examination, computer-assisted histometric measurements were obtained using an automated image analysis system (Image-Pro Plus®, Media Cybernetics, Silver Spring, USA) coupled with a video camera mounted in a light microscope (Leica DM-LB, Leica, Wetzlar, Germany). The measurment parameters were as follows: the cementoenamel junction (CEJ) and the notch were used as reference points (bN) and the histometric parameters included defect height (DH), junctional epithelium (JE), connective tissue attachment (CT), cementum regeneration (NC) and bone regeneration (NB) (Fig. 2).

### Statistical analysis

Histomorphometric recordings from the four sections from each defect were used to calculate the mean score for each animal. Statistical analysis was compared by the Kruskal-Wallis test. The ANOVA Bonferroni method was used to evaluate the statistical significance among the 4 groups (*P*<0.05).

## RESULTS

### Clinical findings

During the postoperative period, healing was uneventful for all animals. There were no signs of inflammation and no wound exposures.

### Histological findings

Histologically, the junctional epithelium migrated apically and inflammatory cell infiltration was minimal in all defect sites. Connective tissue attachment was observed perpendicular to the long axis of the tooth beneath the junctional epithelium. The periodontal ligament was organized with primarily irregular collagen fibers.

**Figure 3:**
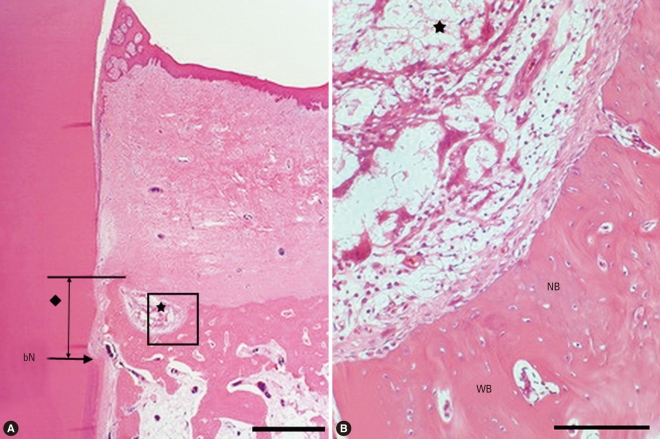
Surgical sections from the calcium phosphate glass cement (CPGC) group. (A) Histologic view of the CPGC group. Most particles were resorbed and new bone was formed above the notch (H&E, ×20; base of reference notch [bN]: arrow; height of new bone: black diamond; CPGC particle: black star; bar = 2 mm). (B) Histologic view of magnified black square area (×200). Osteoblast-like cells were observed around remaining particles. Peripheral new bone was woven bone with isolated osteocytes (H&E, ×200; CPGC particle: black star; NB: new bone; WB: woven bone; bar = 0.1 mm).

The CPGC group showed a large amount of new cementum and new bone formation, more than that seen in the control and CM groups. Resorption of graft material was detectable and several calcium phosphate remnants were surrounded by provisional connective tissue, which would eventually transform into bone. The regenerated bone tissue was embedded and formed within graft materials. The edge of the particles exhibited irregular features and the presence of multinucleated giant cells, indicating the resorption or dissolution process. In the mature new bone portion, osteoclasts observed on the surface of a secondary osteon within the bone trabeculae suggested an active remodeling process. New cementum that was thinner than the original cementum was observed along the root surface, extending to the level of the regenerated bone. A residual collagen membrane was not observed in the CPGC, BCP, or CM groups (Fig. 3).

**Figure 4:**
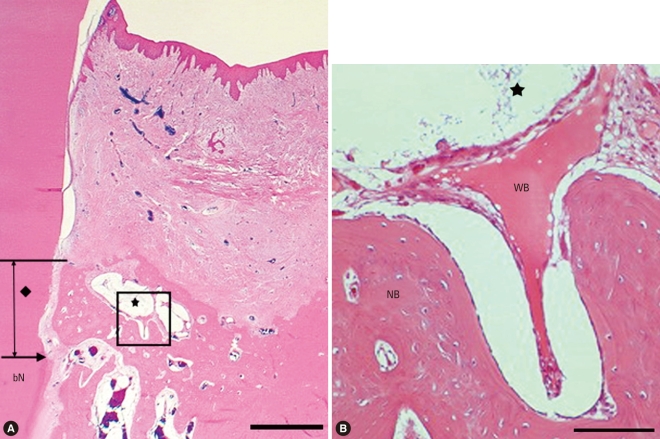
Surgical sections from the biphasic calcium phosphate (BCP) group. (A) Histologic view of BCP group. There were more remaining particles than in the calcium phosphate glass cement (CPGC) group. New bone was formed above the notch (H&E, ×20; base of reference notch [bN]: arrow; the height of new bone: black diamond; BCP particle: black star; bar = 2 mm). (B) Histologic view of magnified black square area (×200). Multi-nucleated giant cells were arranged around particles and woven bone was formed (H&E, ×200; BCP particle: black star; NB: new bone; WB: woven bone; bar = 0.1 mm).

The BCP group showed newly formed woven bone and connective tissue around the remaining HA coated with β-TCP particles. There were no osteoblasts or osteoclasts around BCP particles adjacent to the defect base. However, loose connective tissue including undifferentiated cells and blood vessels was observed around the coronal particles. Regenerated cementum and periodontal ligament space could be observed along the root surface, demonstrating that root resorption or root ankylosis had not occurred (Fig. 4).

**Figure 5:**
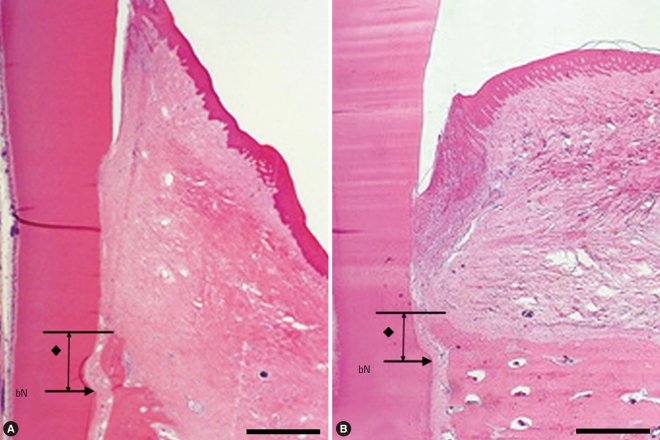
Surgical sections from the collagen membrane (CM) group and the control group. (A) Histologic view of the CM group. A small amount of new bone was formed above the notch. Thick connective tissue was present (H&E, ×20; base of reference notch [bN]: arrow; height of new bone: black diamond; bar = 2 mm). (B) Histologic view of the control group. There was very little new bone above the notch. Long junctional epithelium was observed and inflammatory cell infiltrate was relatively strong (H&E, ×20; base of reference notch [bN]: arrow; height of new bone: black diamond; bar = 2 mm).

The CM group showed some regeneration of bone and cementum occurring along the root surface. Long junctional epithelium was formed more apically than in the other groups. The magnitude of resorption appeared greater in the root surface without cementum than in the root surface covered by new cementum (Fig. 5).

The control group showed only rare regeneration of bone and cementum. A few inflammatory cells were observed at the surgical site. No ankylosis was observed (Fig. 5).

### Histometric findings

The results of histometric analysis are summarized in Table 1. The average defect height was not significantly different among groups.

There were significant differences in new bone height between the CPGC and BCP groups and the CM and control groups. In addition, new cementum height was higher in the CPGC and BCP groups than in the CM and control groups. Epithelial attachment was highest in the CM group. The CPGC group showed less epithelial attachment than the BCP group, but this difference was not significant.

## DISCUSSION

This study was designed to investigate bone formation and periodontal regenerative abilities of CPGC compared with BCP. For this purpose, we used 1-wall intrabony defects, which implies that the defect does not heal by itself during the lifetime of the animal. Without treatment (control group), less than 20% of the bone was regenerated. Therefore, this model is useful for the evaluation of the regenerative effect of bone substitutes in dogs.

The height of new bone formation was 1.90 mm in the CPGC group and 2.42 mm in the BCP group; this difference was not statistically significant. Likewise, the height of new cemetum was 1.34 mm in the CPGC group and 1.70 mm in the BCP group, which lacked statistical significance. These findings suggest that CPGC has an effect similar to BCP on the regeneration of periodontal tissues, including new bone and cementum. Previous studies found similar effects of CPGC on new bone formation. Nery et al. [11] reported that the combination of calcium (Ca) and phosphate (P) in a ceramic implant enhanced repopulation of cells, new periodontal tissue attachment, and bone regeneration within the space of a periodontal osseous defect. In other words, the activities of osteoblasts were promoted by calcium and phosphate ions which were separated during the process of melting the graft material. Both the CPGC and BCP used in this study were a combination of calcium and phosphate and thus would contribute to new bone formation and periodontal regeneration in the bony defects. However, the Ca/P ratio is different in CPGC than in BCP. The Ca/P ratio of CPGC is about 0.6 and that of BCP ranges between 1.50 and 1.67 [12]. This difference may result in different resorption rates of the two graft materials, because Ca^2+^ ions act as a network modifier in glass and secure bond strength between particles. As the Ca^2+^ ion portion increases, the resorption rate of glass decreases [13]. Therefore, when comparing Figs. 3,4, the number of remaining particles was smaller with CPCG than BCP after 8 weeks.

Generally, the barrier membrane is surgically removed after 4-6 weeks in guided tissue regeneration or guided bone regeneration. Connective tissue and bone regeneration may then occur within the bony lesion protected by the barrier [14]. Likewise, CPGC provides sufficient space for optimal wound stability while being resorbed after 8 weeks. This indicates that CPGC has the proper physical properties to serve as a graft material.

It should be mentioned that CPGC may be potentially useful in hard tissue surgery because of its solubility behavior, since its solubility may be controlled by altering its chemical composition [15]. Again, CPG, which is the basis of CPGC synthesis, is associated with the release of biologically therapeutic molecules. CPG can dissolve some elements, oxides, or biological molecules that are insoluble or poorly soluble in glasses of other materials and crystalline compounds. Therefore, CPGC has the ability to not only promote new bone formation and periodontal regeneration but also work synergistically when synthesized with various materials. Many animal studies have demonstrated the ability of CPGC to function as a carrier of growth factor or human recombinant bone morphogenetic protein and have reported positive results [16,17]. More studies are necessary to further clarify the potential applications of CPGC.

The findings of this study provide limited evidence of the excellent physical properties associated with CPCG. Future areas of interest might include determination of the resorption period and resorption rate of this compound. In addition, there should be further research to demonstrate the application of CPGC for widespread use.

## Figures and Tables

**Table 1:**
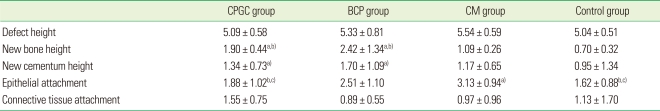
Comparison of histometric analysis among groups (mean ± SD in mm).

CPGC: calcium phosphate glass cement, BCP: biphasic calcium phosphate, CM: collagen membrane. ^a)^Statistically significant difference from control (*P*<0.05). ^b)^Statistically significant difference from CM group (*P*<0.05). ^c)^Statistically significant difference from BCP group (*P*<0.05).

## References

[1] Parikh SN (2002). Bone graft substitutes in modern orthopedics. Orthopedics.

[2] Ahlmann E, Patzakis M, Roidis N, Shepherd L, Holtom P (2002). Comparison of anterior and posterior iliac crest bone grafts in terms of harvest-site morbidity and functional outcomes. J Bone Joint Surg Am.

[3] Kurashina K, Kurita H, Wu Q, Ohtsuka A, Kobayashi H (2002). Ectopic osteogenesis with biphasic ceramics of hydroxyapatite and tricalcium phosphate in rabbits. Biomaterials.

[4] Jarcho M, Kay JF, Gumaer KI, Doremus RH, Drobeck HP (1977). Tissue, cellular and subcellular events at a bone-ceramic hydroxylapatite interface. J Bioeng.

[5] Rabalais ML, Yukna RA, Mayer ET (1981). Evaluation of durapatite ceramic as an alloplastic implant in periodontal osseous defects. I. Initial six-month results. J Periodontol.

[6] Gao H, Tan T, Wang D (2004). Effect of composition on the release kinetics of phosphate controlled release glasses in aqueous medium. J Control Release.

[7] Bagambisa FB, Joos U, Schilli W (1990). Interaction of osteogenic cells with hydroxylapatite implant materials in vitro and in vivo. Int J Oral Maxillofac Implants.

[8] Kim YK, Yun PY, Lim SC, Kim SG, Lee HJ, Ong JL (2008). Clinical evaluations of OSTEON as a new alloplastic material in sinus bone grafting and its effect on bone healing. J Biomed Mater Res B Appl Biomater.

[9] Hosono H, Abe Y (1995). Porous glass-ceramics composed of a titanium phosphate crystal skeleton: a review. J Non-Cryst Solids.

[10] Lee BH, Kim MC, Kim KN, LeGeros RZ, Lee YK (2006). Biodegradable bone cement using calcium phosphate glass. Key Eng Mater.

[11] Nery EB, Eslami A, Van Swol RL (1990). Biphasic calcium phosphate ceramic combined with fibrillar collagen with and without citric acid conditioning in the treatment of periodontal osseous defects. J Periodontol.

[12] Bohner M (2001). Physical and chemical aspects of calcium phosphates used in spinal surgery. Eur Spine J.

[13] Bunker BC, Arnold GW, Wilder JA (1984). Phosphate glass dissolution in aqueous solutions. J Non-Cryst Solids.

[14] Murphy KG, Gunsolley JC (2003). Guided tissue regeneration for the treatment of periodontal intrabony and furcation defects. A systematic review. Ann Periodontol.

[15] Dias AG, Lopes MA, Gibson JR, Santos JD (2003). In vitro degradation studies of calcium phosphate glass ceramics prepared by controlled crystallization. J Non-Cryst Solids.

[16] Maus U, Andereya S, Gravius S, Ohnsorge JA, Niedhart C, Siebert CH (2008). BMP-2 incorporated in a tricalcium phosphate bone substitute enhances bone remodeling in sheep. J Biomater Appl.

[17] Wolff KD, Swaid S, Nolte D, Bockmann RA, Holzle F, Muller-Mai C (2004). Degradable injectable bone cement in maxillofacial surgery: indications and clinical experience in 27 patients. J Craniomaxillofac Surg.

